# Targeting myeloid cells in the tumor microenvironment enhances vaccine efficacy in murine epithelial ovarian cancer

**DOI:** 10.18632/oncotarget.3597

**Published:** 2015-03-14

**Authors:** Anm Nazmul H. Khan, Nonna Kolomeyevskaya, Kelly L. Singel, Melissa J. Grimm, Kirsten B. Moysich, Sayeema Daudi, Kassondra S. Grzankowski, Sashikant Lele, Lourdes Ylagan, Gill A. Webster, Scott I. Abrams, Kunle Odunsi, Brahm H. Segal

**Affiliations:** ^1^ Department of Medicine, Roswell Park Cancer Institute, Buffalo, NY, USA; ^2^ Department of Gynecologic Oncology, Roswell Park Cancer Institute, Buffalo, NY, USA; ^3^ Department of Immunology, Roswell Park Cancer Institute, Buffalo, NY, USA; ^4^ Department of Cancer Prevention and Control, Roswell Park Cancer Institute, Buffalo, NY, USA; ^5^ Department of Pathology, Roswell Park Cancer Institute, Buffalo, NY, USA; ^6^ Innate Immunotherapeutics, Auckland, New Zealand; ^7^ Department of Medicine, University at Buffalo School of Medicine, Buffalo, NY, USA

**Keywords:** ovarian cancer, macrophages, myeloid-derived suppressor cells, vaccination

## Abstract

Epithelial ovarian cancer (EOC) is typically diagnosed at advanced stages, and is associated with a high relapse rate. Patients in remission are ideal candidates for immunotherapy aimed at cure or prolonging disease-free periods. However, immunosuppressive pathways in the tumor microenvironment are obstacles to durable anti-tumor immunity. In a metastatic syngeneic mouse model of EOC, immunosuppressive macrophages and myeloid-derived suppressor cells (MDSCs) accumulate in the local tumor environment. In addition, resident peritoneal macrophages from non-tumor-bearing mice were highly immunosuppressive, abrogating stimulated T cell proliferation in a cell contact-dependent manner. Immunization with microparticles containing TLR9 and NOD-2 ligands (MIS416) significantly prolonged survival in tumor-bearing mice. The strategy of MIS416 immunization followed by anti-CD11b administration further delayed tumor progression, thereby establishing the proof of principle that myeloid depletion can enhance vaccine efficacy. In patients with advanced EOC, ascites analysis showed substantial heterogeneity in the relative proportions of myeloid subsets and their immunosuppressive properties. Together, these findings point to immunosuppressive myeloid cells in the EOC microenvironment as targets to enhance vaccination. Further studies of myeloid cell accumulation and functional phenotypes in the EOC microenvironment may identify patients who are likely to benefit from vaccination combined with approaches that deplete tumor-associated myeloid cells.

## INTRODUCTION

Epithelial ovarian cancer (EOC) typically is diagnosed at advanced stages. Despite primary surgery and adjuvant chemotherapy, the majority of patients have relapse of disease, highlighting the need for novel therapeutic approaches. Patients in remission with minimal disease burdens are ideal candidates for immunotherapy including vaccination aimed at cure or extending progression-free survival [1]. The critical role of immune surveillance in EOC was demonstrated by correlation of survival with tumor-infiltrating lymphocytes (TILs) [2]. Intraepithelial CD8^+^ TILs and a high CD8^+^/regulatory T cell (Treg) ratio were associated with favorable prognosis in patients with EOC [3]. Tumor cells and macrophages in the tumor microenvironment produce CCL22, which mediates trafficking of Tregs to the tumor and poorer prognosis in EOC [4]. Major obstacles to the development of successful immune therapies include the inability to generate an immune response sufficient to cause tumor rejection and the tumor's ability to evade immune attack [5].

Cancer vaccine technologies aim to induce spontaneous anti-tumor immune responses by enhancing the clonal expansion of tumor antigen-specific cytotoxic T lymphocytes (CTLs) as well as central memory T-cells. However, anti-tumor vaccine efficacy may be limited as a result of several factors, including low immunogenicity of cancer-specific antigens, poor trafficking of effector T cells to the tumor microenvironment, and induction of tolerogenic pathways (e.g., Tregs, PD-1, prostaglandin E2) that inhibit T cell-driven anti-tumor immunity. Furthermore, while surgery can induce innate immune responses that facilitate wound healing, this can also create a local immunosuppressive environment that impedes immunotherapy [6].

There is increasing evidence that specific innate immune populations accumulate in the EOC microenvironment and can impede durable anti-tumor immunity. For example, B7-H4, a ligand for inhibitory co-receptors on T cells, is expressed by tumor-associated macrophages and primary tumor cells, and is implicated in antigenic tolerization and tumor escape [7, 8]. Accumulation of B7-H4-expressing macrophages in the tumor microenvironment can impede T cell responses and correlate with more rapid tumor recurrence in EOC [9, 10]. In addition, myeloid-derived suppressor cells (MDSCs), which are a heterogeneous population of immature myeloid cells that can be induced by tumor cells, inhibit T cell responses and secrete factors (e.g., vascular endothelial growth factor and metalloproteinases) that enhance tumor growth, invasion and metastasis. MDSCs accumulate in the local tumor microenvironment and systemically as a function of disease burden in murine EOC [11]. Importantly, Obermajer et al. [12] showed that MDSCs accumulate in the ascites of patients with advanced EOC, and that their purified CD11b^+^ myeloid cells suppressed T cell proliferation *ex vivo.* Furthermore, changes in the phenotype of tumor-infiltrating dendritic cells (DC) have also been shown to influence EOC progression in mice [13]. Together, these findings show that specific innate immune populations may serve as both potential prognostic markers to predict time to relapse as well as therapeutic targets to enhance anti-tumor immunity in EOC.

Our overall hypothesis is that anti-tumor vaccine efficacy would be enhanced if followed by myeloid cell depletion. MIS416 is a novel microparticle derived from *Propionibacterium acnes* and comprised of immune-stimulatory muramyl dipeptide and bacterial DNA, which signals through NOD-2 and TLR9 receptors, and is capable of inducing DC maturation and cross-presentation that promotes CTL polarization and Th1 immunity [14]. MIS416 is being explored as an immune-based therapy for multiple sclerosis [15]. Since MIS416 induces immunological responses that may be useful as a cancer vaccine adjuvant, we investigated MIS416 in a metastatic syngeneic murine model of EOC. The ovarian tumor cell line used in this model was engineered to express ovalbumin (OVA) as a nominal tumor antigen and transferred naïve OT-I cells were used to evaluate antigen specific CD8^+^ T cell responses. Immunization with MIS416 plus OVA increased the accumulation of transferred OT-I cells in the local tumor microenvironment and systemically, and modestly delayed tumor progression. However, MIS416 vaccination also led to increased peritoneal accumulation of granulocytic MDSCs, which are predicted to impede durable anti-tumor immunity. Although CD11b^+^ myeloid cell depletion by itself had no benefit, sequential immunization followed by myeloid cell depletion led to significant delay in tumor progression compared to vaccination alone. These studies establish the proof of principle that broad myeloid cell depletion can enhance MIS416 vaccine efficacy in EOC. Additional studies of the tumor microenvironment in patients with advanced EOC showed substantial heterogeneity in myeloid cell accumulation and also in their immunosuppressive phenotype, raising the potential for identifying patients who are likely to benefit from targeting tumor-associated myeloid cells to enhance the efficacy of immunotherapy.

## RESULTS

### Resident and tumor-associated peritoneal macrophages in mice suppress T cell proliferation

**Figure 1:**
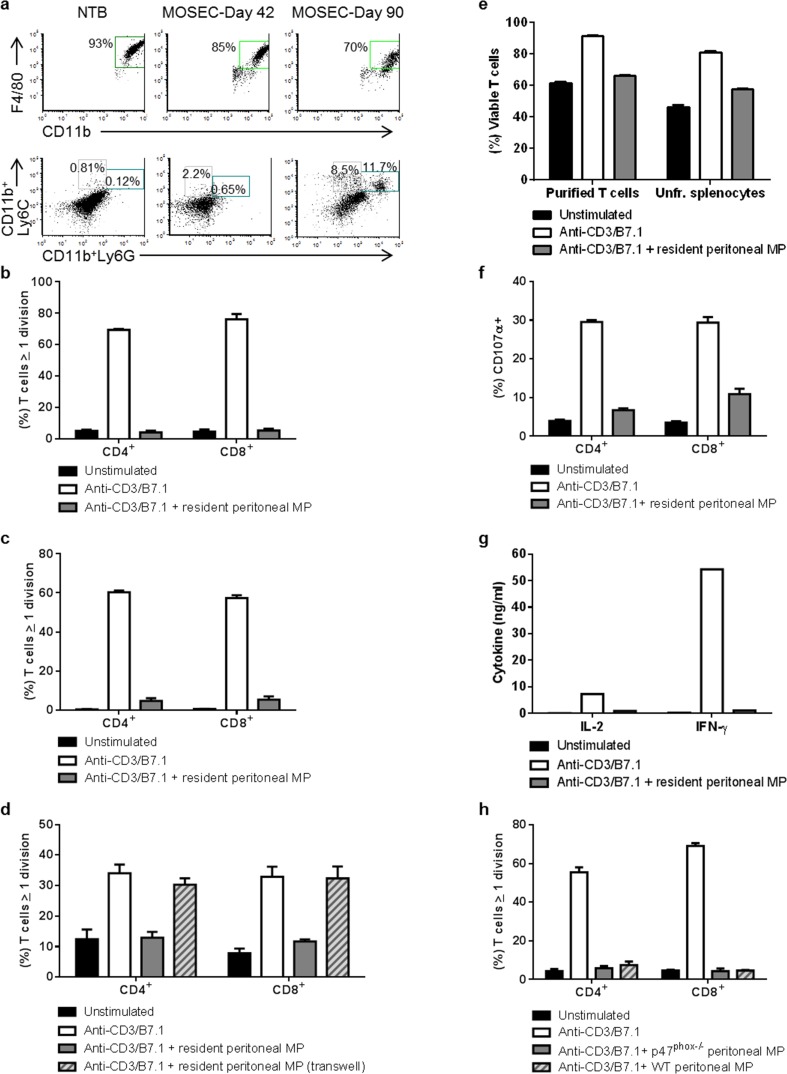
Peritoneal macrophages from non-tumor-bearing and MOSEC-ID8-bearing mice suppress T cell proliferation and activation a) Macrophages (MP) are the predominant peritoneal myeloid cell in non-tumor-bearing (NTB) and MOSEC-ID8-bearing mice. Representative dot-plots showing peritoneal MP (CD11b^+^F4/80^+^), granulocytic MDSCs (CD11b^+^Ly6G^+^Ly6C^low^), and monocytic MDSCs (CD11b^+^Ly6C^+^Ly6G^−^) in NTB and MOSEC-ID8-bearing mice (1 mouse/group) at day 42 and 90. CD11b^+^ populations from total cells were gated to obtain percent of MP, granulocytic and monocytic MDSCs. b-c) Resident peritoneal MP abrogate anti-CD3/B7.1-stimulated CD4^+^ and CD8^+^ T cell proliferation. b) Resident peritoneal MP from NTB mice were co-cultured with CFSE-labeled splenocytes from naïve mice (E:T ratio 1:1) in triplicate in anti-CD3/B7.1-coated 96-well plates. After 72 hours of culture, CD4^+^ and CD8^+^ T cell proliferation was assessed based on CFSE dilution. Data are representative of 3 independent experiments. c) The same approach was used as described in (b), except that splenic T cells column-purified by negative selection were used as targets. Data are representative of 3 independent experiments. d) Resident peritoneal MP mediate suppression of CD4^+^ and CD8^+^ T cell proliferation in a cell-cell contact-dependent manner. Similar to b, resident peritoneal MP from NTB mice and CFSE-labeled splenocytes were co-cultured in an anti-CD3/B7.1-coated 24-well plate transwell system, and after 72 hours T cell proliferation was assessed. Data are representative of 2 independent experiments. e) Resident peritoneal MP mediate suppression of CD4^+^ and CD8^+^ T cell proliferation independent from apoptosis. Similar to c, purified peritoneal MP from NTB mice were either co-cultured with CFSE-labeled purified T cells or unfractionated splenocytes from naïve mice. Total T cell populations were gated to determine apoptotic death based on annexin V and 7-AAD labeling. Percent viable T cells (annexin V^−^/7-AAD^−^) from triplicates in different groups are presented. f) Activation of T cells was suppressed by resident peritoneal MP. Expression of CD107α on CD4^+^ and CD8^+^ T cells was assessed from (c) to determine the percent of T cell activation. g) MP suppressed the level of IL-2 and IFN-γ in cell-free supernatants analyzed from the same experiment (c). The average cytokine levels from duplicate samples per group measured by standard ELISA are presented. These experiments were repeated at least two times with similar results. h) Peritoneal MP from MOSEC-ID8-bearing mice suppress T cell proliferation independent of NADPH oxidase (NOX2). Column-purified peritoneal MP from wild-type [WT-MP] and NOX2-deficient [p47phox^−/−^-MP] mice at day 90 after MOSEC-ID8 administration were evaluated for their effects on anti-CD3/B7.1 stimulated CD4^+^ and CD8^+^ T cell suppression. Data are representative of 3 mice per group from 3 separate experiments.

In a metastatic model of murine EOC using intraperitoneal (i.p.) administration of syngeneic mouse ovarian surface epithelial cancer cells (MOSEC-ID8), we previously observed that granulocytic MDSCs (CD11b^+^Ly6G^+^Ly6C^low^) accumulated in the peritoneum as a function of tumor burden, and suppressed stimulated T cell proliferation, while non-myeloid (CD11b^−^) peritoneal cells from tumor-bearing mice either incompletely suppressed or had no effect on stimulated T cell proliferation *ex vivo* [11]. Prior studies have also shown that resident tissue macrophages in mice reversibly suppress T cell proliferation [16]. We therefore evaluated the effects of peritoneal macrophages from both non-tumor-bearing (NTB) and MOSEC-ID8-bearing mice on stimulated T cell proliferation and activation. In NTB naïve mice, peritoneal myeloid cells were >90% macrophages (CD11b^+^F4/80^+^) (Fig. 1a). In MOSEC-ID8-bearing mice, macrophages constituted the predominant population of peritoneal myeloid cells, with variable numbers of granulocytic MDSCs and monocytic MDSCs (CD11b^+^Ly6C^+^Ly6G^−^) detected at both early (day 42 after tumor challenge) and advanced (day 90) disease stages (Fig. 1a). Similar to MDSCs that accumulate during tumor progression, resident peritoneal macrophages from NTB mice abrogated anti-CD3/B7.1-stimulated CD4^+^ and CD8^+^ T cell proliferation. This suppressive effect of peritoneal macrophages was observed when co-cultured with unfractionated splenocytes (Fig. 1b) and with purified splenic CD4^+^ and CD8^+^ T cells from NTB mice (Fig. 1c and Supplemental Fig. 1). We next evaluated whether resident macrophage-mediated T cell suppression was contact-dependent using the transwell system, and found that the absence of cell-cell contact abrogated the suppressive effect of peritoneal macrophages from NTB mice (Fig. 1d).

The abrogation of T cell proliferation can occur through a number of mechanisms, including induction of apoptosis of T cells. To evaluate this possibility, unfractionated splenocytes or purified splenic T cells were co-cultured with peritoneal macrophages from NTB mice, and stimulated with anti-CD3/B7.1 for 72 hours. T cell death was assessed by 7-AAD and annexin-V staining. The proportion of viable T cells (7-AAD^−^/Annexin-V^−^) was highest in anti-CD3/B7.1-stimulated T cells in the absence of peritoneal macrophages, and modestly reduced in unstimulated conditions and following stimulation with anti-CD3/B7.1 in the presence of peritoneal macrophages (Fig. 1e and Supplemental Fig. 2). These results suggest that although peritoneal macrophages may augment T cell apoptosis indirectly by abrogating anti-CD3/B7.1 pro-proliferative signaling, the major suppressive effect of peritoneal macrophages is not through induction of T cell death. Co-culture with resident peritoneal macrophages also reduced the proportion of anti-CD3/B7.1-stimulated T cells expressing CD107α (a marker of T cell activation) (Fig. 1f), and suppressed the production of IL-2 and IFN-γ (Fig. 1g). These results support the notion that resident peritoneal macrophages contribute to an immunosuppressive environment by suppressing T cell proliferation and activation.

Similar to resident macrophages in NTB mice, peritoneal macrophages in MOSEC-ID8-bearing mice were highly immunosuppressive. Almost complete inhibition of anti-CD3/B7.1-stimulated T cell proliferation occurred when co-cultured with peritoneal macrophages (CD11b^+^F4/80^+^) isolated from MOSEC-ID8 mice at day 90 after tumor challenge (Fig. 1h). Since the phagocyte NADPH oxidase (NOX2) can have important signaling functions, including antigen presentation [17, 18], we evaluated whether NOX2 in macrophages was relevant to their T cell suppressive function. Peritoneal macrophages from MOSEC-ID8-bearing NOX2-deficient (p47*^phox−/−^*MP) mice suppressed *ex vivo* T cell proliferation to a similar degree as MOSEC-ID8-bearing wild-type (WT) macrophages (Fig. 1h). Together, these observations demonstrate that both resident and tumor-associated peritoneal macrophages may contribute to the immunosuppressive milieu in the EOC tumor microenvironment, which may be a barrier to anti-tumor immunity.

### MIS416 vaccination augments antigen-specific CTL expansion, but promotes accumulation of granulocytic MDSCs in murine EOC

We next evaluated whether vaccination would mitigate the immunosuppressive environment in EOC and prolong survival. We used MOSEC-IE9 cells, which are engineered to express OVA as a tumor-associated antigen and adoptive transfer of OVA-specific OT-I cells [19]. This model enabled us to track the effect of MIS416 vaccination on tumor progression and antigen-specific CD8^+^ T cell responses to a model antigen. At a time point corresponding to low disease burden (day 30 after MOSEC-IE9 administration), mice were adoptively transferred with naïve OT-I cells, followed by immunization with MIS416 mixed with OVA (days 31 and 38). In two separate experiments, MIS416 immunization extended the median time to tumor progression requiring euthanasia by approximately 2 weeks.

**Figure 2:**
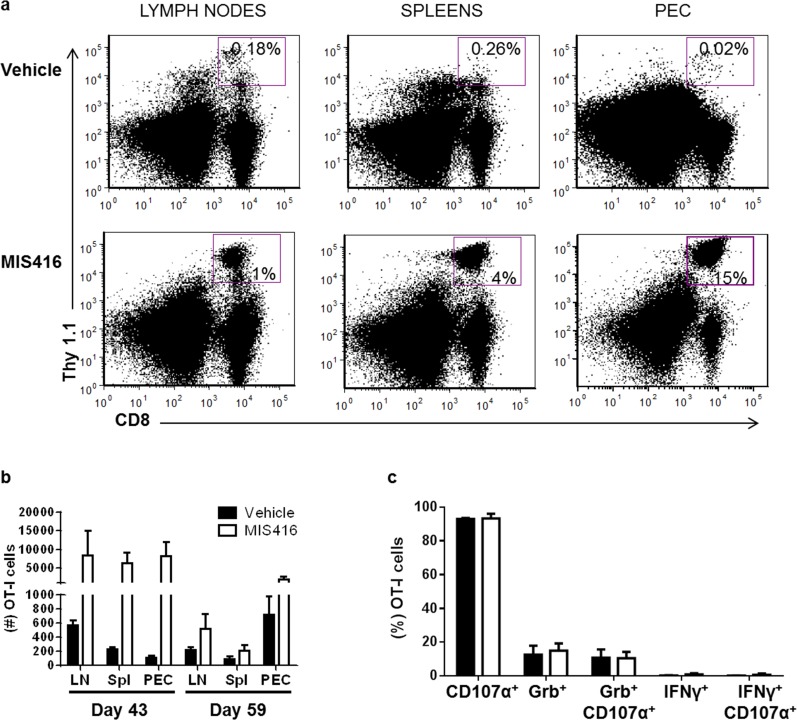
Vaccination with MIS416 increases accumulation of OT-I cells in the tumor microenvironment and systemically in ovarian tumor-bearing mice At day 30 after i.p. MOSEC-IE9 administration, mice were adoptively transferred with naïve OT-I cells, followed by immunization with MIS416 mixed with OVA (days 31 and 38) or vehicle mixed with OVA (control). Lymph node cells (LN), splenocytes (Spl) and PECs harvested at day 43 and 59 after tumor administration were analyzed for OT-I cell accumulation (n = 3 mice per group per time point). a and b) MIS416 administration increased accumulation of OT-I cells in the local peritoneal environment and systemically at day 43 after tumor challenge. a) Representative dot plots of MIS614 and vehicle groups showing accumulation of CD8^+^Thy1.1^+^ (%) cells in different compartments. Total cells were gated to obtain the percent positive cells for CD8 and Thy1.1. b) On day 59 after tumor administration, OT-I cell accumulation had substantially waned in MIS416-vaccinated mice. Data are representative of 3 mice per group from 2 separate experiments. c) MIS416 vaccination did not significantly affect the proportion of peritoneal OT-I cells expressing the activation marker CD107α or granzyme B (Grb) at day 59 after tumor administration (n = 3 mice per group). Interferon (IFN)-γ^+^ OT-I cells were ≤2% in MIS416-vaccinated and control groups. Thus immunization with MIS416 increased the accumulation of transferred OT-I cells in the local tumor microenvironment and systemically, but did not affect their phenotype based on the markers evaluated.

Since the MIS416 vaccine was modestly protective, we next determined its effects on CTL and myeloid cell responses to elucidate the mechanisms behind the lack of more durable anti-tumor responses. Tumor-bearing mice treated with MIS416 vaccine or vehicle on days 31 and 38 (n = 3 per treatment group per time point) were sacrificed on days 43 or 59 in relation to MOSEC-IE9 administration, corresponding to early and more advanced tumor burden, respectively. At day 43, MIS416 vaccine administration led to a dramatic increase in OT-I cell accumulation in peritoneal exudate cells (PEC), tumor-draining lymph nodes (TDLN), and spleens (Fig. 2a). However, at day 59, OT-I cell accumulation had substantially waned in MIS416-vaccinated mice, but remained higher than vehicle-treated mice (Fig. 2b). The proportion of peritoneal OT-I cells expressing granzyme B, CD107α, or dual expression of these markers was similar in MIS416-vaccinated compared to control mice, and the proportion expressing IFN-γ was ≤ 2% in both groups (Fig. 2c). These results show that MIS416 vaccine dramatically increases the accumulation of OT-I cells both in the local tumor microenvironment and systemically in tumor-bearing mice without significantly altering the effector phenotypes of these cells. However, the effect on OT-I cell expansion was short-lived, and the overall effect of vaccination on time to euthanasia was modest.

**Figure 3:**
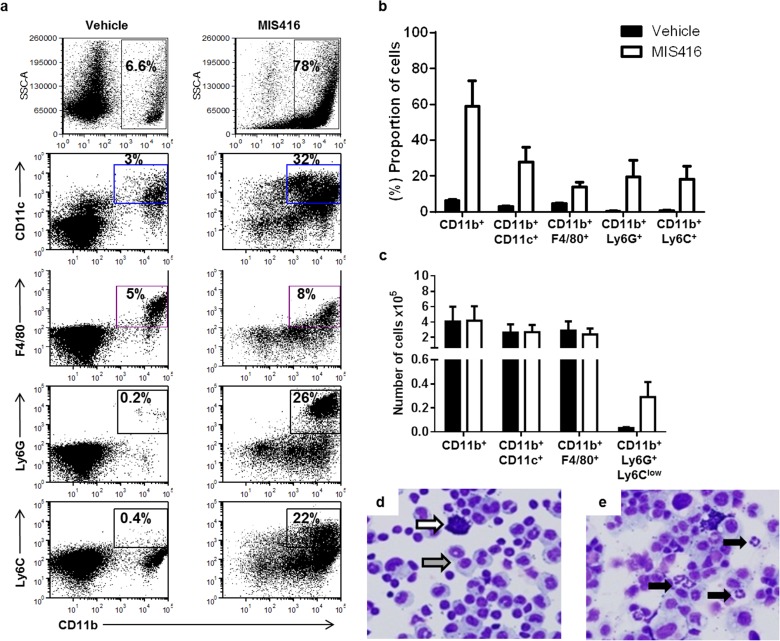
MIS416 vaccination increases granulocytic MDSC accumulation in the peritoneum of ovarian tumor-bearing mice At day 30 after i.p. MOSEC-IE9 administration, mice were adoptively transferred with naïve OT-I cells, followed by immunization with MIS416 mixed with OVA (days 31 and 38) or vehicle mixed with OVA (control). a and b) MIS416 administration led to an increased accumulation of the proportion of myeloid cells and of multiple myeloid subsets in the peritoneum at day 43 after tumor administration. Representative dot plots of a single mouse from each group (a) and average data from 3 mice/group (b) show substantial increase in the proportion of total myeloid (CD11b^+^) cells and specific myeloid subsets: DCs (CD11b^+^CD11c^+^), macrophages (MP; CD11b^+^F4/80^+^), granulocytic (CD11b^+^Ly6G^+^) and monocytic (CD11b^+^Ly6C^+^) cells in PECs isolated from MIS416-vaccinated compared to vehicle-treated mice. Total cells from SSC and FSC plots were gated to obtain (%) CD11b^+^, CD11b^+^CD11c^+^, CD11b^+^F4/80^+^, CD11b^+^Ly6G^+^ and CD11b^+^Ly6C^+^ cells. Data are representative of 2 separate experiments. c) MIS416 vaccination led to increased peritoneal accumulation of granulocytic MDSCs (CD11b^+^Ly6G^+^Ly6C^low^) on day 43. The peritoneal accumulation of myeloid cell populations in individual mice was calculated from the total number of PECs based on hemocytometer counts and the proportion of (%) myeloid subsets based on surface expression. MIS416-vaccinated mice had increased accumulation of granulocytic MDSCs (CD11b^+^Ly6G^+^Ly6C^low^), but not DCs or MP. Data are from 3 mice/group. d and e) Cytology of unfractionated PECs confirmed the accumulation of cells with granulocytic morphology in MIS416-vaccinated mice (e), while these cells were virtually absent in control mice (d). White arrow, tumor cells; grey arrow, macrophages; black arrows, granulocytic cells. Data are representative of 3 mice/group from 2 separate experiments.

Since EOC progression is associated with the accumulation of immunosuppressive myeloid cells, we next evaluated the effect of MIS416 vaccine on local and systemic myeloid cell accumulation and immunosuppressive phenotype in tumor-bearing mice. MIS416 administration led to an increased proportion of myeloid cell (CD11b^+^) accumulation consisting of multiple myeloid subsets in the peritoneum at day 43 after tumor administration (Figs. 3a and b). A substantial decrease in the proportion of non-myeloid (CD11b^−^) cell accumulation (containing mostly tumor cells as confirmed by cytology) after MIS416 vaccination, indicated an anti-tumor effect by MIS416 (Fig. 3a). The accumulation of myeloid cell populations in the peritoneal cavities of individual mice was calculated from the total number of PECs (based on hemocytometer counts) and the proportion of myeloid subsets based on multi-color flow cytometry (average from 3 mice per group). MIS416-vaccinated mice had increased accumulation of granulocytic cells (CD11b^+^Ly6G^+^Ly6C^low^), but not macrophages (MP; CD11b^+^F4/80^+^) or DC (CD11b^+^CD11c^+^) on day 43 (Fig. 3c). Cytology of unfractionated PECs confirmed the accumulation of cells with granulocytic morphology in MIS416-vaccinated mice, while these cells were virtually absent in control mice (Figs. 3d and e).

**Figure 4:**
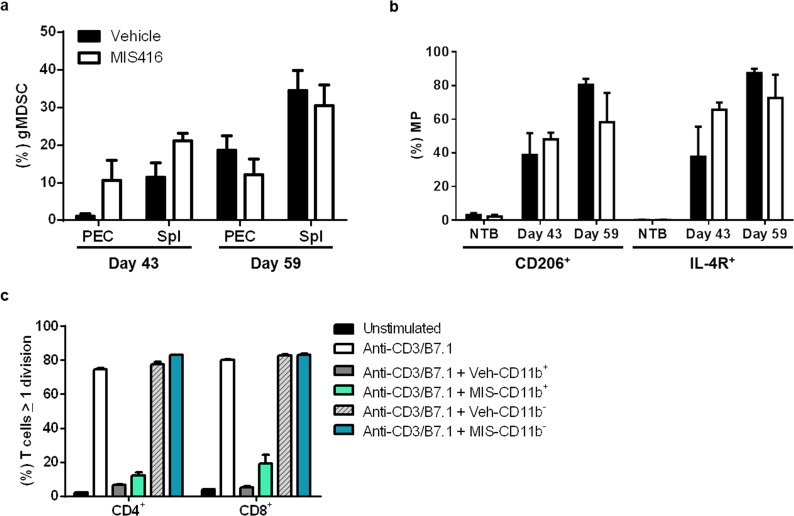
MIS416 vaccination does not affect the immunosuppressive properties of myeloid cells including macrophages and granulocytic MDSCs in MOSEC-IE9-bearing mice a) The proportion of (%) granulocytic MDSCs (gMDSC; CD11b^+^Ly6G^+^Ly6C^low^) accumulation in the local tumor microenvironment and systemically was increased after MIS416 vaccination on day 43, but by day 59, no significant difference between MIS416-vaccinated and vehicle groups was observed. b) The proportion of peritoneal macrophages (MP; CD11b^+^F4/80^+^) expressing M2 markers, CD206 and IL-4R, increased in tumor-bearing mice, but was not consistently affected by MIS416 administration (NTB, non-tumor-bearing). c) Peritoneal myeloid cells from MIS416- and vehicle-treated tumor-bearing mice suppressed T cell proliferation. Column-purified CD11b^+^ PECs harvested at day 59 after MOSEC-IE9 administration (corresponding to 21 days after 2^nd^ MIS416 vaccination) were assessed for suppression of anti-CD3/B7.1-stimulated CD4^+^ and CD8^+^ T cell proliferation. Non-myeloid (CD11b^−^) PECs from both groups of mice, which predominantly contained tumor cells and lymphocytes, were not suppressive. Data are representative of 3 mice per group from 3 separate experiments.

MIS416 vaccination increased the proportion of granulocytic cells in the peritoneum and spleen on day 43 (Fig. 4a). By day 59, no significant difference in the proportion of systemic or local MDSCs between the MIS416 vaccine and vehicle groups was observed. In addition, macrophage (MP; CD11b^+^F4/80^+^) subset analysis for the M2 markers, CD206 and IL-4R, showed that in NTB mice, only a small percentage (≤ 5%) of peritoneal macrophages expressed M2 markers. In contrast, a substantial proportion of peritoneal macrophages expressed M2 markers on days 43 and 59 after tumor administration (Fig. 4b). We did not observe a consistent effect of MIS416 vaccine on the proportion of peritoneal macrophages expressing M2 markers in tumor-bearing mice (Fig. 4b). Peritoneal myeloid cells (CD11b^+^) containing macrophages and granulocytic MDSCs from both MIS416-vaccinated and vehicle-treated mice on day 59 abrogated stimulated T cell proliferation while the non-myeloid fraction had no significant effect (Fig. 4c). These results show that early effects of MIS416 vaccination include decreases in the tumor burden, and increases in the proportion of myeloid cells and total granulocytic MDSCs in the local tumor microenvironment (day 43). However, at a later time point (day 59) corresponding to more advanced tumor burden, the effect on granulocytic MDSCs by MIS416 vaccine was no longer detectable. These results also show that MIS416 vaccination does not alter the immunosuppressive capacity of macrophages and granulocytic MDSCs in peritoneal myeloid cells at this time point.

Since macrophages and myeloid DCs have a shared lineage, we evaluated the effect of MIS416 vaccine on splenic accumulation of these cells. We used standard markers for macrophages (CD11b^+^F4/80^+^) and myeloid DCs (CD11b^+^CD11c^+^), and also evaluated dual expression of the classical macrophage and DC markers (CD11b^+^F4/80^+^CD11c^+^). CD11c is expressed by several macrophage populations, including those associated with epithelial and inflammatory macrophages [20-22], and has generally been associated with an M1 phenotype [23-25]. MIS416 vaccine increased the proportion of splenic DCs at day 59 after tumor challenge, but did not significantly affect the proportion of macrophages or dual expressing F4/80^+^CD11c^+^ cells (Supplemental Figure 3a). We also observed an increase in the proportion of splenic macrophages and DCs expressing DEC-205 (Supplemental Figure 3b), which mediates antigen internalization and cross-presentation [26, 27]. MIS416 vaccine did not alter the proportion of splenic DCs expressing CLEC9A (data not shown), a receptor that recognizes damaged cells and promotes antigen uptake and cross-presentation [28, 29].

### Myeloid cell depletion enhances MIS416 vaccine efficacy against ovarian tumor

**Figure 5:**
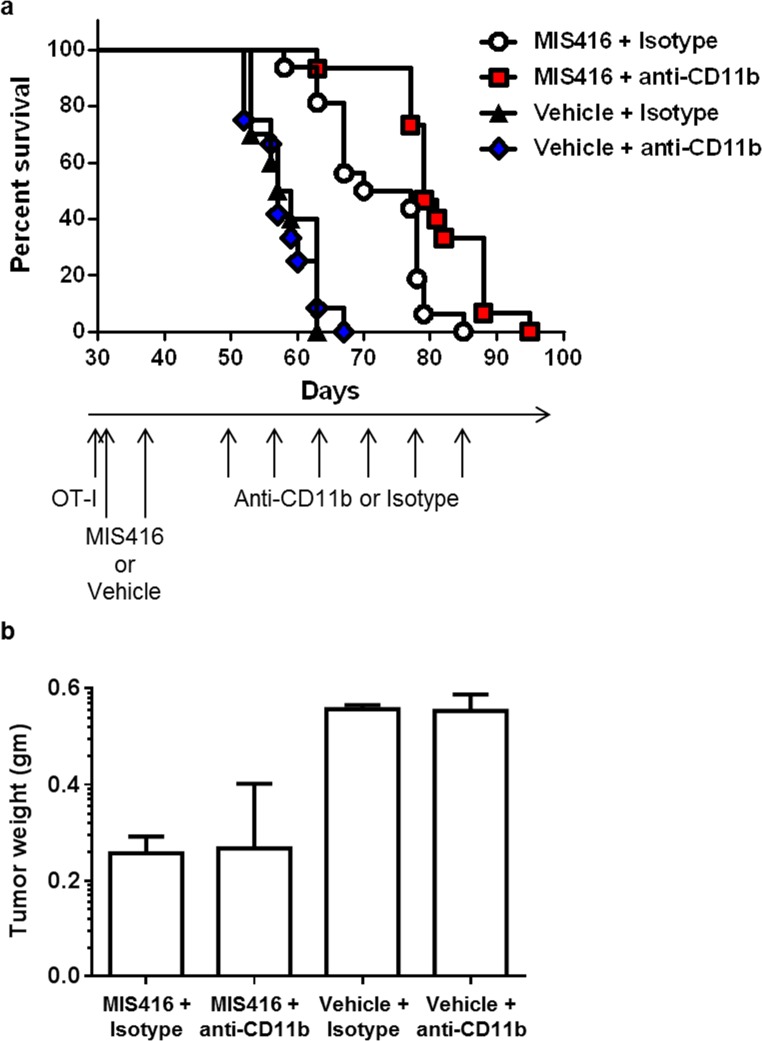
Myeloid cell depletion enhances the efficacy of MIS416 immunization in ovarian tumor-bearing mice a) Adoptive transfer of OT-I cells was performed in MOSEC-IE9-bearing mice at day 30. Vaccination (MIS416 plus OVA) or control (vehicle plus OVA) treatments were administered on days 31 and 38. Beginning on day 50, mice were administered weekly anti-CD11b mAb or control IgG (isotype) for a maximum of 6 weeks, and monitored for tumor progression requiring euthanasia. Time to euthanasia was displayed by Kaplan-Meier plots (n = 16 mice per group). MIS416 vaccination followed by isotype significantly prolonged time to euthanasia compared to treatment with vehicle followed by isotype (log-rank, p < 0.0001). MIS416 vaccination followed by anti-CD11b mAb treatment led to significantly prolonged survival compared to MIS416 vaccination followed by isotype (p = 0.0013). In the absence of prior MIS416 vaccination, anti-CD11b mAb had no benefit. b) Similarly treated mice (n = 3 per treatment group) were pre-selected for sacrifice at day 59 after tumor challenge, and visible tumor was removed and weighed. MIS416-vaccinated mice had reduced tumor weight compared with non-vaccinated groups (p = 0.004; adjusted p-value for multiple comparisons = 0.01), while anti-CD11b mAb had no significant effect.

Since MIS416 vaccination enhanced antigen-specific CTL accumulation in the tumor microenvironment and systemically while also promoting the accumulation of immunosuppressive myeloid cells, we reasoned that vaccination followed by non-selective myeloid depletion using anti-CD11b mAb to target both tumor-associated macrophages and MDSCs might prolong vaccine-induced anti-tumor immunity. The primary endpoint was time to euthanasia based on pre-specified morbidity criteria. Anti-CD11b mAb treatment (or isotype) was begun ~20 days after adoptive transfer of naïve OT-I cells and 12 days after the last MIS416 vaccination. This schedule of MIS416 and anti-CD11b mAb administration was selected to enable vaccine-induced DC activation and antigen presentation, and expansion of transferred OT-I cells prior to subsequent myeloid cell depletion (Fig. 2). Anti-CD11b mAb administration to non-immunized mice led to modest depletion of all subsets of peritoneal myeloid cells (macrophage, DC and MDSC) at day 59 after tumor challenge, with the strongest effect being a ~4-fold depletion of granulocytic MDSCs (Supplemental Fig. 4). Administration of anti-CD11b mAb following MIS416 immunization led to a similar level of myeloid depletion, with the major effect on granulocytic MDSCs. Anti-CD11b mAb alone or in combination with MIS416 vaccine did not affect effector OT-I cell expansion or the proportion of endogenous T cells at day 59 after tumor administration (data not shown). MIS416 vaccination alone significantly increased time to tumor progression requiring euthanasia (log-rank p < 0.0001) (Fig. 5a). Although anti-CD11b mAb by itself had no effect on tumor progression, the strategy of MIS416 vaccination followed by anti-CD11b mAb significantly prolonged survival compared to vaccination followed by isotype (log-rank p = 0.0013) (Fig. 5a). In mice pre-selected for sacrifice at day 59 after tumor challenge (n = 3 per treatment group), MIS416 vaccination led to reduced tumor weight, while anti-CD11b mAb treatment had no significant effect at this time point (Fig. 5b). Together these data show that: (i) MOSEC tumor growth leads to an accumulation of immunosuppressive macrophages and MDSCs in the peritoneal tumor microenvironment; (ii) MIS416 vaccination modulates both host myeloid cells and OT-I cell accumulation in the tumor microenvironment and systemically and prolongs survival of tumor-bearing mice; (iii) Sequential vaccination followed by myeloid cell depletion significantly extends time to tumor progression requiring euthanasia.

### Heterogeneity in ascitic myeloid cell accumulation and immunosuppressive phenotype in patients with advanced EOC

**Figure 6:**
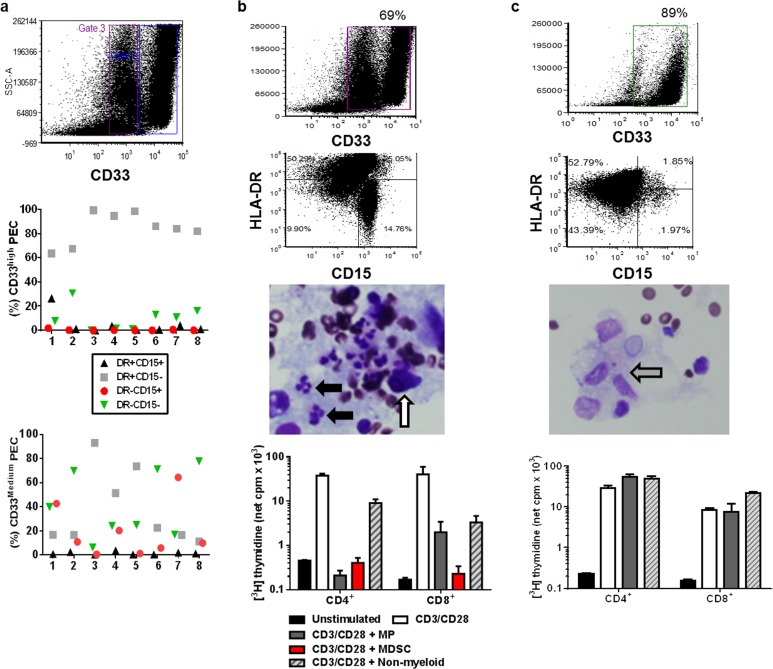
Heterogeneity in ascitic myeloid cell populations and immunosuppressive activity in patients with advanced EOC Myeloid cells from ascites collected at the time of primary surgery from 8 patients with EOC were evaluated. a) Gating on CD33^high^ (gate 4) and CD33^medium^ (gate 3) myeloid populations, the proportion of macrophages (MP; DR^+^CD15^−^), granulocytic (DR^−^CD15^+^), myelomonocytic (DR^+^CD15^+^), and immature myeloid (DR^−^CD15^−^) cells was determined. b and c) There were dramatic differences in the composition and immunosuppressive phenotype of ascitic myeloid cells. As examples, while the peritoneal myeloid fraction of patient 1 (b) contained a mixed population of mature MP, granulocytic cells, mixed myelomonocytic cells and immature cells, a paucity of granulocytic cells was present in the ascites of patient 2 (c). Cytology of ascites from these patients was consistent with the flow cytometry data (white arrow, tumor cell; grey arrow, macrophages; black arrows, granulocytic cells). Both mature MP and non-MP myeloid cells (MDSC-rich fraction) from patient 1 suppressed stimulated T cell proliferation to basal levels, while peritoneal MP from patient 2 did not suppress stimulated T cell proliferation. Non-myeloid PECs from all samples had modest or no T cell suppressive activity.

Immunosuppressive myeloid cells are known to accumulate in the ascites of patients with EOC [9, 10, 12]. Based on our data from murine EOC, we undertook a more detailed analysis of macrophages and MDSCs in ascites of patients with EOC and evaluated their functional properties. Myeloid cells from ascites collected at the time of primary surgery from 8 patients were evaluated. Macrophages were defined based on CD33^+^DR^+^CD15^−^ expression, granulocytic cells were defined based on CD33^+^DR^−^CD15^+^ expression, a mixed myelomonocytic lineage was defined by CD33^+^DR^+^CD15^+^ expression, and immature myeloid cells were defined based on lack of expression of macrophage or granulocytic markers (CD33^+^DR^−^CD15^−^). CD33^medium^ and CD33^high^ populations were observed in all patients, with the proportion of each population varying among patients. Mature macrophages principally segregated in the CD33^high^ group, virtually all granulocytic cells were CD33^medium^, and immature myeloid cells were observed in both CD33^medium^ and CD33^high^ groups. There was substantial inter-patient variability in the proportion of myeloid cell populations in ascites (Fig. 6a). This variability was most obvious in the granulocytic cell population, which made up a significant population of myeloid cells in the ascites of some patients and was virtually absent in others.

We next evaluated the immunosuppressive function of ascitic macrophages and MDSCs. Since MDSCs are a heterogeneous population of immature cells, and expression of surface markers can overlap with mature myeloid cells, we applied stringent criteria to FACS purification of macrophages, requiring high surface expression of 2 markers (CD14 and DR) expressed at late stages of macrophage differentiation. The remaining myeloid cell population was defined as MDSCs if they exhibited immunosuppressive function. The immunosuppressive function of myeloid PECs in ascites was defined based on their ability to suppress proliferation of purified anti-CD3/CD28-stimulated allogeneic T cells from a normal volunteer, as described [30]. All T cells were purified from the same normal donor. We found striking inter-patient variability in the immunosuppressive properties of myeloid PECs from the 8 patients tested. As illustrated in Fig. 6b, both mature macrophages and non-macrophage myeloid cells (MDSC-rich fraction) from patient 1 suppressed stimulated T cell proliferation to basal levels. In contrast, peritoneal macrophages from patient 2 did not suppress stimulated T cell proliferation (Fig. 6c). The sort-purified MDSC population from patient 2 contained insufficient cell numbers to include in this experiment. Non-myeloid PECs from all samples had modest or no T cell suppressive activity. Together, these results raise the potential for distinct populations of ascitic myeloid cells that can suppress T cell immunity in the tumor microenvironment in patients with advanced EOC.

## DISCUSSION

Our results in murine models and in patients with EOC support the hypothesis that immunosuppressive MDSCs accumulate in the local tumor environment and also systemically as a function of disease burden, suppressing T cell immunity, which likely facilitates tumor progression. Immunization with MIS416, a novel adjuvant comprising NOD-2 and TLR9 stimulatory ligands, significantly prolonged survival in tumor-bearing mice. We expect that the major anti-tumor effect of MIS416 vaccine in our model was driven by the increased accumulation of OT-I cells in the tumor microenvironment. However, this increased accumulation of activated OT-I cells was short-lived, dissipating by 3 weeks after completion of immunization. MIS416 vaccine increased the accumulation of splenic DCs and the proportion expressing DEC-205, which may enhance cross-presentation. However, MIS416 vaccine also led to increased local and systemic accumulation of immunosuppressive granulocytic MDSCs, which are likely to be a barrier to durable vaccine-induced immunity. The strategy of vaccination followed by broad myeloid cell depletion using anti-CD11b mAb significantly delayed tumor progression compared to vaccination alone, demonstrating that myeloid depletion can enhance vaccine efficacy.

An advantage of anti-CD11b mAb treatment is that it targets both immunosuppressive macrophages as well as MDSCs that accumulate in the tumor microenvironment. Even with incomplete myeloid cell depletion, we observed a modest but statistically significant effect in enhancing vaccine efficacy. The tumor microenvironment can alter the biology of myeloid cells, including the acquisition of immunosuppressive and pro-angiogenic properties that promote tumor progression [31]. Cui et al. [32] showed that MDSCs can increase the stemness of cancer cells by microRNA101 signaling, and increased MDSC density and tumor microRNA101 expression predicted poor survival in patients with ovarian cancer. Several strategies have been used to deplete or alter the phenotype of tumor-associated myeloid cells. As examples, administration of liposomal clodronate-depleted macrophages, but not neutrophils or natural killer cells, reduced tumor progression and VEGF expression in murine EOC (reviewed in [33]). Selective delivery of pro-apoptotic peptides to tumor-associated M2 macrophages improved survival in tumor-bearing mice [34]. CXCR4 antagonist-expressing oncolytic virotherapy decreased tumor growth in murine EOC, and was associated with reduced accumulation of myeloid cells and Tregs [35]. Consistent with the notion of myeloid cells as an obstacle to successful anti-cancer treatment, Ahn et al. [36] showed that myeloid depletion with anti-CD11b mAb enhanced radiation sensitivity of administered colon tumor in mice. Trabectedin, an alkylating agent with anti-tumor activity, also targets the monocyte/macrophage lineage in tumor-bearing models and in treated patients [37]. The effects of myeloid depletion on tumor control varied in different models. For example, Tomihara et al. [19] showed that granulocytic myeloid cells (CD11b^+^Gr-1^+^) in ascites of ovarian tumor-bearing mice actually enhanced antigen-specific immunity and promoted regression of tumor. This highlights the need for further studies delineating the role of myeloid subsets on tumor progression to enable more specific targeting of this approach.

Resident peritoneal macrophages from unstimulated NTB mice were highly immunosuppressive, completely abrogating anti-CD3/B7.1-stimulated T cell proliferation. This suppression was contact-dependent, but independent of NADPH oxidase. These results are consistent with prior studies showing that resident tissue macrophages reversibly suppress T cell proliferation [16]. Vascular leukocytes are recruited to the ovarian cancer microenvironment, suppress T cell activation and promote neovascularization, drive tumor progression in murine models, and are promising targets for therapeutic depletion [38-41]. Our results build on these findings by showing that resident peritoneal macrophages in NTB mice contribute to the immunosuppressive peritoneal microenvironment. We speculate that resident peritoneal macrophages may facilitate tumor growth during early disease, prior to the induction of tumor-elicited MDSCs and M2 macrophages that are associated with more advanced disease. If this model is correct, then targeting peritoneal macrophages may enhance the effects of immunotherapy initiated in patients with EOC with minimal disease burden. A related question that merits further investigation is whether in the setting of minimal residual tumor burden following optimal surgery and chemotherapy, resident peritoneal macrophages promote disease relapse.

Based on these results in murine EOC, we undertook an analysis of macrophages and MDSCs in the ascites of patients with advanced EOC. Our principal goal was to evaluate the proportion and immunosuppressive phenotype of mature macrophages versus other myeloid cells in the EOC microenvironment. We found substantial heterogeneity in the proportion of ascitic myeloid cell subsets and in their immunosuppressive properties. Mature macrophages (CD33^+^DR^+^CD15^−^) were generally the predominant myeloid subset, while the proportion of granulocytic cells (CD33^+^DR^−^CD15^+^) was highly variable. Prior studies showed that MDSCs accumulated in the ascites of patients with advanced EOC, and that purified CD11b^+^ myeloid cells suppressed T cell proliferation *ex vivo* [12]. However, this study did not distinguish the effect of ascitic macrophages versus MDSCs on suppression of T cell responses. In addition, accumulation of the suppressive B7-H4-expressing macrophage subset in the tumor environment was associated with more rapid tumor recurrence in EOC [9, 10]. Solid tumor cells, including primary human ovarian cancer cells, express B7-H4, and neutralizing antibodies targeting B7-H4 enhanced anti-tumor T cell immunity and delayed tumor progression [7]. Furthermore, expression of the M2 marker CD163 on macrophages in ascites samples was inversely associated with relapse-free survival in patients with EOC [42]. Ascitic cytokine profiles have been correlated with outcome in patients with EOC [43, 44]. Simpson-Abelson et al. [45] showed that ascites from patients with EOC inhibited T cell receptor-induced NF-kB and NFAT signaling in tumor-associated T cells. Moreover, T cells from ascites of EOC patients were less responsive to antigenic stimulation than circulating lymphocytes [46]. Together, these findings point to the tumor microenvironment in EOC as being both pro-inflammatory and suppressive of T cell immunity.

Our results support a model in which peritoneal macrophages contribute to a locally immunosuppressive environment in the absence of tumor, and innate immune responses during EOC further abrogate cellular immunity. Consistent with this notion, myeloid cell depletion enhanced anti-tumor vaccine efficacy in murine EOC after adoptive transfer of naïve OT-I cells. The OT-I system used in our studies has been widely used in tumor-bearing experiments [47, 48], but has important limitations. The OVA antigen is not a “self” antigen spontaneously expressed on tumor cells, and T cell responses to endogenous tumor antigens likely do not have the same affinity and proliferative capacity as OT-I lymphocytes. Another limitation is that we did not evaluate different doses, routes of administration, and schedules of MIS416 vaccination and anti-CD11b treatments. Optimizing these factors may lead to more sustained efficacy in delaying tumor progression. Finally, we intentionally used a broad myeloid depletion strategy to target both macrophages and granulocytic cells; a limitation of this approach is that we cannot delineate the effects of specific myeloid cell populations on vaccine-induced anti-tumor immunity.

In humans, EOC leads to an accumulation of a peritoneal myeloid cell population consisting of mature macrophages, immature myeloid cells and granulocytic cells with variable immunosuppressive phenotypes. Further studies are warranted to derive and validate prognostic signatures for EOC that model the proportion of tumor-associated myeloid cell subsets and their immunosuppressive function. In addition, such studies may identify patients with EOC who are likely to benefit from vaccination combined with approaches that target tumor-associated myeloid cells.

## MATERIALS AND METHODS

### Mice

Female C57BL/6, OVA-specific TCR transgenic OT-I/Rag^−/−^ (Jackson Laboratory, Bar Harbor, ME), and NADPH oxidase-deficient (p47*^phox−/−^*) mice [49] were used at 6 – 8 weeks of age. All mice were maintained under specific pathogen free conditions at the animal care facility at Roswell Park Cancer Institute (RPCI) and used in compliance with all relevant laws and institutional guidelines under a protocol approved by the RPCI Animal Care and Use Committee.

### Mouse ovarian surface epithelial cancer (MOSEC) cells

The MOSEC-ID8 line (provided by Dr. Paul Terranova, University of Kansas Medical Center, Kansas City, KS) was derived from epithelial ovarian cells harvested from female C57BL/6 mice that were passaged *in vitro* [50]. Intraperitoneal (i.p.) injection of clonal lines established from late passage epithelial cells from syngeneic tumors in mice results in ascites and peritoneal implants that mimic the human disease [11, 50]. The OVA-expressing MOSEC-IE9 cell line was generated as described [19], and provided to us by Dr. Tahiro Shin (University of Texas Health Sciences Center, San Antonio, TX). MOSEC-ID8 and MOSEC-IE9 cells were cultured in RPMI 1640 media with heat-inactivated FBS (10%), L-glutamine (2 mM), HEPES (25 mM), sodium pyruvate (1 mM), 2-mercaptoethanol (50 μM), penicillin/streptomycin (100 μg/ml) and non-essential amino acids.

### Tumor administration

Mice were administered i.p. MOSEC-ID8 or MOSEC-IE9 cells (5 – 10 × 10^6^ cells in PBS), and were monitored daily for 100 days by trained animal care staff blinded to treatment regimens. Moribund mice were euthanized based on the decisions of animal care staff using pre-specified criteria (abdominal distention, lethargy or inability to ambulate). Pre-selected groups of tumor-bearing mice were sacrificed prior to the onset of morbidity for immunologic endpoints.

### Adoptive transfer of OT-I cells

On day 30 after MOSEC-IE9 administration, all tumor-bearing mice underwent adoptive transfer of naïve OT-I lymphocytes (3 × 10^6^ cells/0.2 ml PBS/mouse) by retro-orbital injection. MHC class-I-restricted OVA-antigen specific OT-I cells were used to evaluate the antigen specific CD8^+^ T cell responses after MIS416 vaccination in MOSEC-IE9 bearing mice. Lymph nodes (inguinal, popliteal, brachial, axillary, periaortic, and mesenteric) harvested from OT-I mice were homogenized in sterile conditions. Single lymph node cell suspension was prepared in PBS and purity of OT-I cells (>90%) was confirmed by flow cytometry with anti-CD8 and anti-Thy 1.1 mAb prior to injection.

### Generation of anti-CD11b mAb

Anti-CD11b mAb was generated from ascites of SCID mice after i.p. administration of M1/70 hybridoma (DSHB, University of Iowa, Iowa City, IA) in the Laboratory Animal Research facility at RPCI. The ascites was heat-inactivated and filter-sterilized before *in vivo* administration. *In vivo* titration experiments were conducted in non-tumor-bearing (NTB) and MOSEC-IE9-bearing mice using different volumes of ascites (25 – 200 μl) to measure depletion of CD11b^+^ cells (macrophages, myeloid DCs and neutrophils) in the peritoneum and spleen. Based on >70 % depletion of myeloid cells, anti-CD11b mAb (50 μl) was selected for therapeutic depletion studies. Depletion of myeloid cells was confirmed in the tumor microenvironment by flow cytometry.

### MIS416 vaccination and anti-CD11b mAb treatment

MOSEC-IE9-bearing mice were assigned into 4 groups: 1) MIS416 and anti-CD11b mAb; 2) PBS and anti-CD11b mAb; 3) MIS416 and IgG isotype; 4) PBS and IgG isotype. MIS416 (5.5 mg/ml) or PBS was mixed with OVA solution (180 μg/ml) at 1:1 ratio, and 200 μl per mouse was administered subcutaneously on days 31 and 38 in relation to tumor administration. Beginning on day 50, mice were treated with i.p. anti-CD11b mAb (50 μl ascitic fluids in 150 μl PBS/mouse) or isotype IgG (50 μg in 200 μl PBS/mouse) weekly for 6 weeks or until sacrificed. This treatment schedule of anti-CD11b was chosen to provide sufficient time for effector T cell activation after MIS416 vaccination.

### Immunological analysis in mice

Following sacrifice of mice, peritoneal exudate cells (PECs) were collected by peritoneal lavage with PBS (5 – 8ml, containing 1% FBS and 0.5 mM EDTA). PECs were subjected to RBC lysis with ACK buffer, followed by washing. Tumor-draining lymph nodes (TDLN) and spleens were also collected at harvest, and single cell suspensions were subjected to RBC lysis with ACK buffer, followed by washing. Isolated PECs, splenocytes, and TDLN cells were either used within 24h of harvest for flow cytometry and functional studies or frozen in liquid nitrogen in media containing 20% FBS and 5% DMSO. To evaluate cellular morphology, PECs from each group of mice were analyzed microscopically by Diff-Quick-stained cytospins (Fisher Scientific, Kalamazoo, MI).

Flow cytometry analysis was conducted on a FACScan (Becton Dickinson, Franklin Lakes, NJ). Forward scatter versus side scatter gating was set to include all non-aggregated cells from at least 20,000 events collected per sample. Data were analyzed using FCS Express 4. Fc receptors were blocked with anti-mouse CD16/CD32 antibodies (BD Biosciences, San Jose, CA). PE-Ly6G and -CD8, FITC-Ly6C (BD Biosciences), APC-CD11b, Pacific Blue-CD4, eFlour 450-F4/80 (eBioscience, San Diego, CA), APC-DEC-205, PE-CLEC9A, and PE/Cy7-CD11c (Biolegend, San Diego, CA) anti-mouse mAb, and respective isotype controls were used. Total cells were gated to obtain the percent of myeloid (CD11b^+^), macrophage (CD11b^+^F4/80^+^), DC (CD11b^+^CD11c^+^) and MDSCs (CD11b^+^Ly6G^+^ and CD11b^+^Ly6C^+^) based on specific isotype controls. After gating on CD11b^+^ cells, the proportion of granulocytic MDSCs (Ly6G^+^Ly6C^low^) and monocytic MDSCs (Ly6C^+^Ly6G^−^) was determined. The peritoneal accumulation of total myeloid cell populations in individual mice was calculated from the total number of PECs based on hemocytometer counts and the proportion of (%) myeloid subsets based on surface expression. Effector OT-I cells were analyzed using surface staining for PerCP-CD8 (Biolegend), APC-Cy7-Thy 1.1 and PECy7-CD107α (BD Biosciences), in addition to intracellular staining for PE-Granzyme B (eBioscience) and APC-IFN-γ (BD Biosciences) anti-mouse mAb. Cells were stimulated with SIINFEKL (3 μM for 1h), followed by incubation with Brefeldin A (300 μg/ml for 4h) for intracellular staining. Cells were fixed and permeabilized using the Cytofix/Cytoperm kit following manufacturer's instructions (eBioscience).

Myeloid cell-mediated immunosuppression was evaluated based on suppression of stimulated T cell proliferation in well-established co-culture experiments, as we described [11]. Peritoneal myeloid cells and macrophages from tumor-bearing mice were column-purified with anti-CD11b and anti-F4/80 magnetic beads, respectively, using autoMACS according to the manufacturer's protocol (Miltenyi Biotec Inc., Auburn, CA). Following column separation, the purity of cell fractions was analyzed microscopically by Diff-Quick-stained cytospins (Fisher Scientific) and by flow cytometry (~90% purity). Resident peritoneal macrophages were harvested from NTB mice by peritoneal lavage and column-purified with anti-CD11b or anti-F4/80. Unfractionated splenocytes or splenic T cells column-purified by negative selection (Pan T Cell Isolation Kit, Miltenyi Biotec Inc.) from NTB C57BL/6 female mice were used as naïve T cell targets. Following RBC lysis and washing, unfractionated splenocytes or purified T cells were incubated with 5 μM carboxyfluoresceindiacetate succinimidyl ester (CFSE; Invitrogen, Grand Island, NY) in PBS for 8 min as previously described [11]. Cells (2.5 × 10^5^ cells/well) were cultured in triplicate in 96-well plates pre-coated with anti-CD3 (10 μg/ml) mAb (BD Biosciences) and B7.1 (0.5 μg/ml) (R&D Systems Inc., Minneapolis, MN). Equal numbers of magnetically separated CD11b^−^, CD11b^+^ or F4/80^+^ cells isolated from tumor-bearing mice were added. After 72 hours of co-culture, cells were collected, labeled with anti-CD4 and anti-CD8 mAb, and analyzed by flow cytometry. The proliferation of CFSE-labeled CD4^+^ and CD8^+^ T cells was evaluated by quantification of CFSE dilution. T cell populations from forward/side scatter plots were gated to obtain CD4^+^ and CD8^+^ cells (based on isotype controls) and those cells were gated on histograms to determine proliferation based on CFSE dilution. The primary endpoint was the proportion of CFSE-loaded CD4^+^ and CD8^+^ T cells undergoing ≥ 1 replication. To evaluate apoptotic death of stimulated T cells, cells were stained with APC-Annexin-V and 7AAD (Apoptosis detection kit, eBioscience) after 72 hours of co-culture. At 72 hours, the expression of CD107α on CD4^+^ and CD8^+^ T cells was analyzed by flow cytometry, and the concentrations of IL-2 and IFN-γ in cell-free supernatants were measured by ELISA.

To evaluate whether peritoneal macrophage-mediated suppression of T cell proliferation was contact-dependent, transwell assays were performed. The myeloid cell functional assay, described above in 96-well plates, was modified with a five-fold increase in the number of cells and plated in 24-well plates using the transwell system (VWR International, Bridgeport, NJ). PECs collected from NTB C57BL/6 female mice were purified with anti-CD11b magnetic beads using autoMACS according to the manufacturer's protocol (Miltenyi Biotec Inc.). Splenocytes from NTB mice (2 × 10^6^ cells/well) were plated in the wells of the 24-well companion plate in contact with pre-coated stimulus (anti-CD3 and B7.1), while equal numbers of CD11b^+^ PECs were added in the chamber of the transwell cell culture insert (0.4 μm). After 72 hours of co-culture, cells were collected and analyzed by flow cytometry to measure the proliferation of CFSE-labeled CD4^+^ and CD8^+^ T cells based on quantification of CFSE dilution staining as described.

### Myeloid cells in ascites of patients with newly diagnosed advanced EOC

Ascites (50 ml) was collected for research at the time of primary surgery in patients with newly diagnosed stage III EOC under an IRB-approved protocol. All subjects signed informed consent prior to surgery. PECs, isolated from ascitic fluid by centrifuge (500g for 10 min at 4^0^C), were subjected to RBC lysis with ACK buffer, followed by washing. PECs were either used within 24h of harvest for flow cytometry and functional studies or frozen in liquid nitrogen in media containing 20% FBS and 5% DMSO. PE-Cy5-CD33, PE-CD14 (Beckman Coulter, Brea, CA), BV 412-CD11b (Biolegend), FITC-HLA-DR (BD Biosciences), anti-human mAb and respective isotype controls were used to analyze surface molecule expression and sorting of human PECs. In addition, PE-Cy7-HLA-DR (BD Biosciences), APC-CD14, and FITC-CD15 (Invitrogen) anti-human mAb were used to evaluate the proportion of macrophage and granulocytic cells in PECs from EOC patients.

To evaluate the immunosuppressive properties of peritoneal myeloid cells, PECs were FACS-sorted to isolate macrophages (CD11b^+^CD33^+^CD14^+^DR^+^) and granulocytic cells (CD11b^+^CD33^+^CD14^−^DR^−^CD15^+^). The purity of the post-sort cell population was confirmed by flow cytometry (>90%). Normal donor allogeneic CD4^+^ and CD8^+^ T cells were used as responders in co-culture experiments, as described [30]. T cells were purified with anti-CD4 and anti-CD8 magnetic beads using autoMACS according to the manufacturer's protocol (Miltenyi Biotec Inc.), and preserved in liquid nitrogen with 20% FBS and 5% DMSO in complete media. Freshly isolated PECs (macrophages, granulocytic cells or non-myeloid CD11b^−^CD33^−^ cells) from patients were incubated in triplicate in 96-well round-bottom plates for 4 days with equal numbers (1 × 10^5^) of normal donor T cells (CD4^+^ and CD8^+^). CD3/CD28 Dynabeads (2 μl) (Invitrogen) were added to each well to activate T cell proliferation. T cell proliferation was measured by [^3^H]-thymidine (1 μCi per well) incorporation for the final 18 hours of culture. Results are expressed as net counts per minute (cpm) [average cpm from mixed cultures of T cells with PECs in presence of CD3/CD28 - (average cpm from parallel cultures without T cells in presence of CD3/CD28 + cpm from T cells cultures only without CD3/CD28)].

### Statistical analysis

Time to euthanasia was plotted using Kaplan-Meier curves and analyzed using the log-rank method. Comparisons between two groups were assessed by the Mann-Whitney test, and the Kruskal-Wallis test was used for multiple group comparisons. Statistical analysis was performed using Graph Pad Prism 6 software.

## SUPPLEMENTARY MATERIAL, FIGURES



## Abbreviations

EOC: epithelial ovarian cancer
MDSCs: myeloid-derived suppressor cells
TILs: tumor-infiltrating lymphocytes
NTB: non-tumor-bearing
TDLN: tumor-draining lymph nodes
PECs: peritoneal exudate cells
MOSEC: mouse ovarian surface epithelial cancer.
