# Supplementary material for: Targeting myeloid cells in the tumor microenvironment enhances vaccine efficacy in murine epithelial ovarian cancer
Source: Oncotarget. 2015 Mar 14;6(13):11310–26. doi: 10.18632/oncotarget.3597 (PMC4484458; doi:10.18632/oncotarget.3597)
Supplement: Supplementary file 1 [file oncotarget-06-11310-s001.pdf]

Targeting myeloid cells in the tumor microenvironment enhances vaccine efficacy in murine epithelial ovarian cancer

Supplementary Material

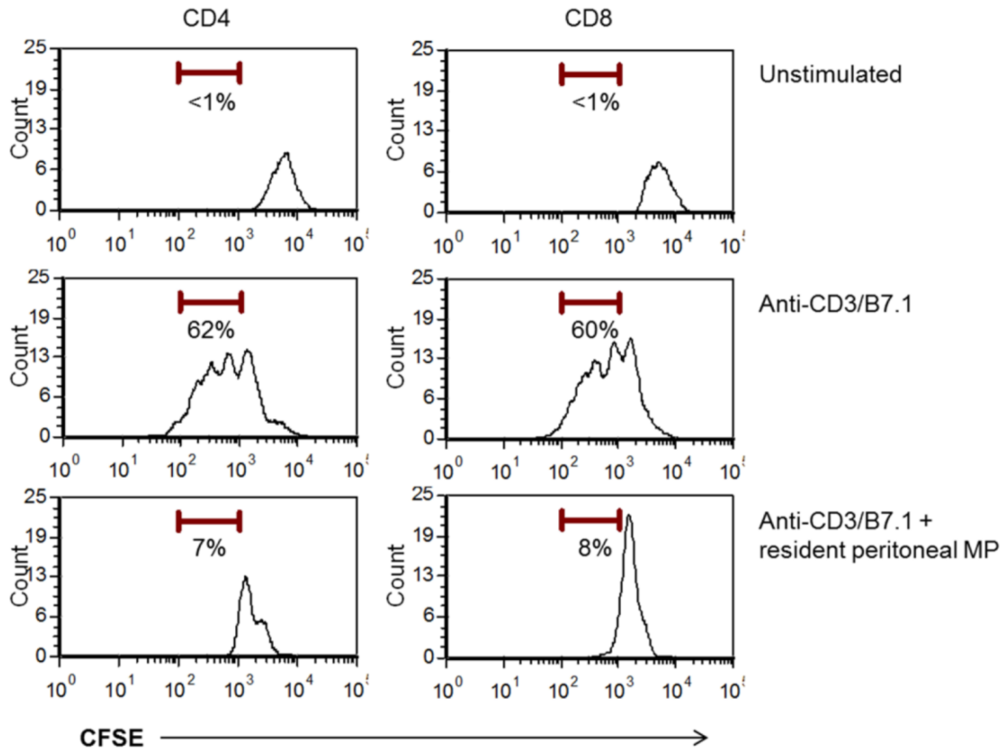

Supplemental Figure 1: Representative histograms show resident peritoneal macrophages (MP) abrogate anti-CD3/B7.1-stimulated CD4 and CD8 T cell proliferation. Column-purified peritoneal MP (F4/80$^+$) from NTB mice were co-cultured with purified T cells from naïve mice (E:T ratio 1:1) in anti-CD3/B7.1-coated 96-well plates, as described in Fig. 1C. After 72 hours of culture, CD4$^+$ and CD8$^+$ T cell proliferation was assessed based on CFSE dilution. CFSE-labeled and unstimulated T cells were used as negative control. Percent positive T cells undergoing ≥ 1 division are shown.

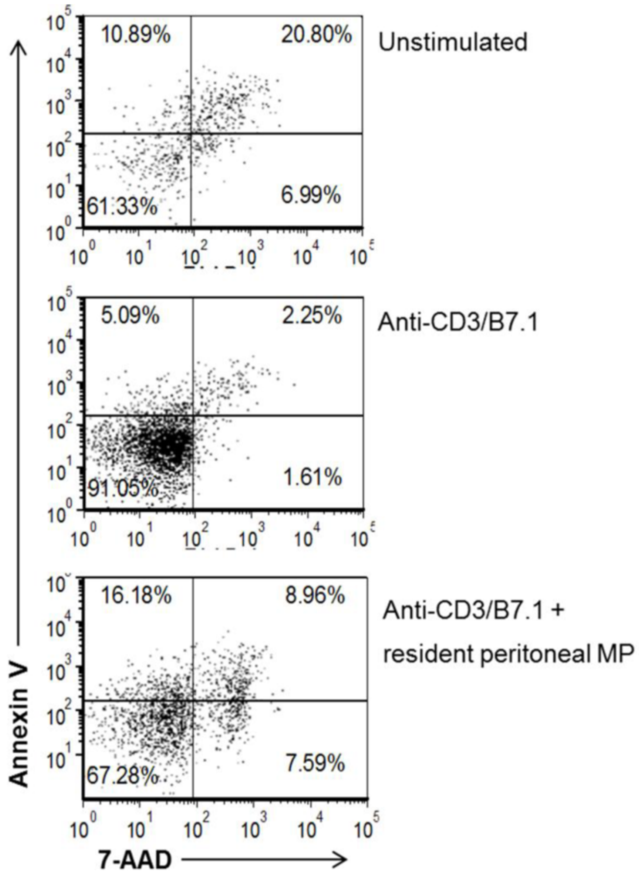

Supplemental Figure 2: Effect of resident peritoneal macrophages (MP) on viability of stimulated T cells. Splenic T cells from NTB mice were purified by negative selection, labeled with CFSE, and incubated as follows: unstimulated; anti-CD3/B7.1 stimulation; and anti-CD3/B7.1 stimulation with addition of column-purified resident peritoneal MP (F4/80^{+}) from NTB mice (E:T ratio 1:1). After 72 hours of culture, total T cell populations were gated based on forward/side scatter, and cell death was assessed by annexin V and 7-AAD staining. The proportion of viable T cells (7-AAD^{-}Annexin-V^{-}) was highest in anti-CD3/B7.1-stimulated T cells, and modestly reduced in unstimulated conditions and following stimulation with anti-CD3/B7.1 in the presence of peritoneal MP. Representative plots show that the suppressive effect of peritoneal MP on stimulated T cell proliferation (as shown in Fig. 1) is not primarily driven by induction of T cell apoptosis. Results are representative of three separate experiments.

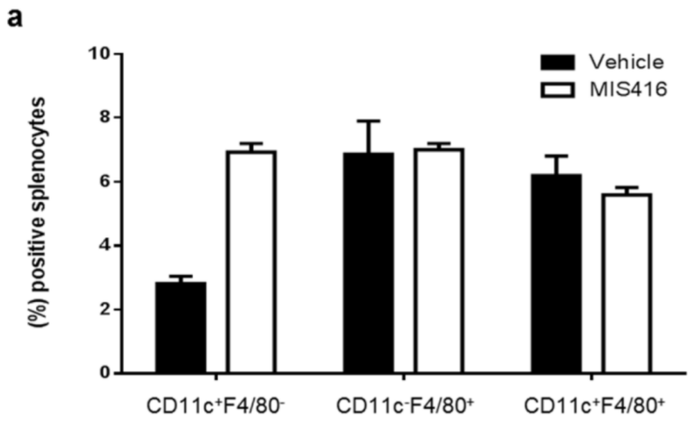

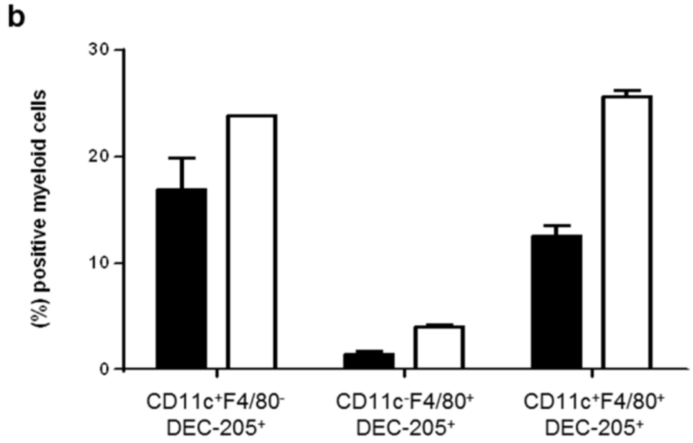

Supplemental Figure 3: MIS416 increases the proportion of splenic DCs and DEC-205-expressing cells in ovarian tumor-bearing mice. Mice were administered i.p. MOSEC-IE9, followed by adoptive OT-I transfer (day 30), MIS416/OVA or vehicle/OVA treatment (days 31 and 38), and were sacrificed on day 59. Splenocytes isolated from mice were analyzed by flow cytometry. Total cells were gated to obtain $CD11c^{+}F4/80^{-}$, $CD11c^{-}F4/80^{+}$, and $CD11c^{+}F4/80^{+}$ cells (A); and those cells were further gated to obtain the proportion of DEC-205-expressing populations (B). Two separate experiments showed similar results (2 mice/group).

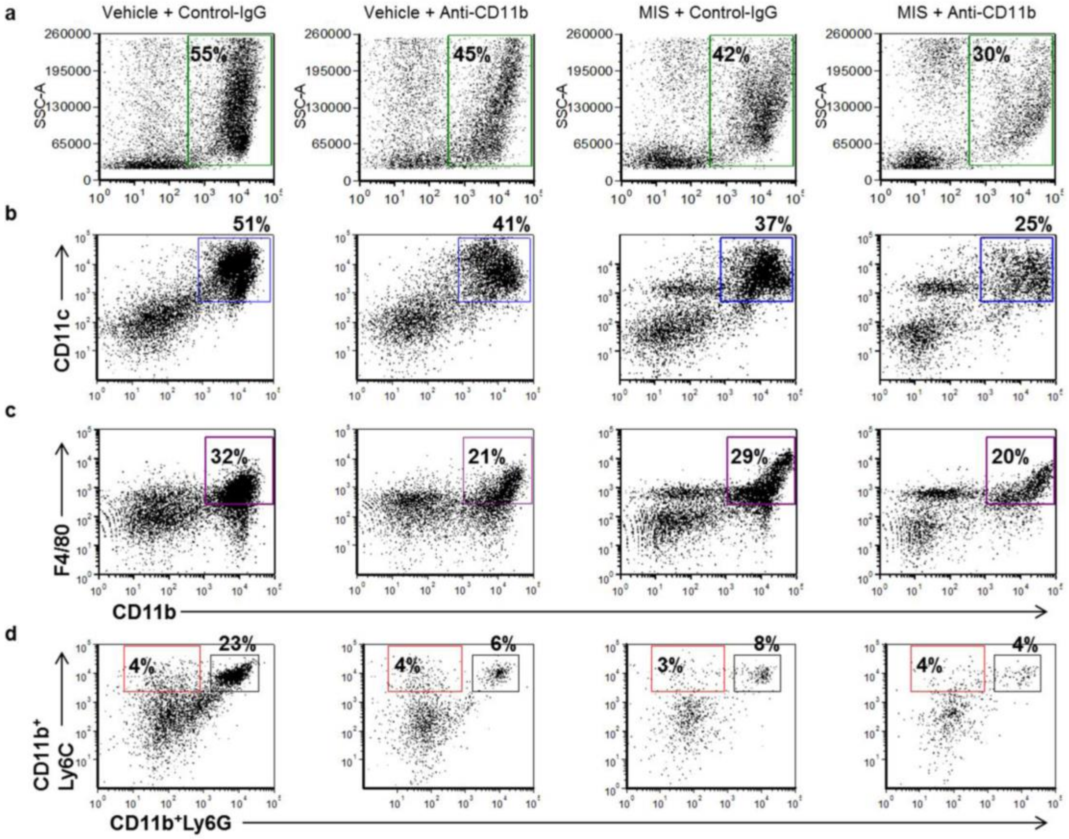

Supplemental Figure 4: Anti-CD11b mAb treatment following MIS416 administration partially depletes peritoneal myeloid cells in ovarian tumor-bearing mice. Mice were administered i.p. MOSEC-IE9, followed by adoptive OT-I transfer (day 30), MIS416/OVA or vehicle/OVA treatment (days 31 and 38), administration of anti-CD11b mAb or isotype-IgG (days 50 and 57), and were sacrificed on day 59. PECs isolated from mice were analyzed by flow cytometry. Representative dot plots (1 mouse/group) show that anti-CD11b mAb treatment partially depleted all myeloid subsets analyzed, with the major effect on the peritoneal granulocytic MDSCs ($CD11b^{+}Ly6G^{+}Ly6C^{low}$). Two separate experiments (3 mice/group) showed similar results.
